# (*E*)-3-Dimethyl­amino-1-(1,3-thia­zol-2-yl)prop-2-en-1-one

**DOI:** 10.1107/S1600536812048817

**Published:** 2012-12-05

**Authors:** Xin-Yu You, Yao-Jie Shi, Luo-Ting Yu

**Affiliations:** a Department of Pharmaceutical and Bioengineering, School of Chemical Engineering, Sichuan University, Chengdu 610065, People’s Republic of China; b State Key Laboratory of Biotherapy and Cancer Center, West China Hospital, West China Medical School, Sichuan University, Chengdu 610041, People’s Republic of China

## Abstract

In the title compound, C_8_H_10_N_2_OS, the 3-(dimethyl­amino)­prop-2-en-1-one unit is approximately planar [give r.m.s. deviation] and the mean plane through the seven non-H atoms makes a dihedral angle of 8.88 (3)° with the thia­zole ring. The carbonyl and ring C=N double bonds adjacent to the carbonyl group are *trans* [N—C—C—O = 172.31 (15) °], while the conformation of the carbonyl and propene double bonds is *cis* [O—C—C—C = 2.2 (2)°]. In the crystal, short C—H⋯N and C—H⋯O inter­actions together with C—H⋯π inter­actions generate a three-dimensional network.

## Related literature

For the biological activity of enaminone derivatives, see: Zeng (2010 ▶).
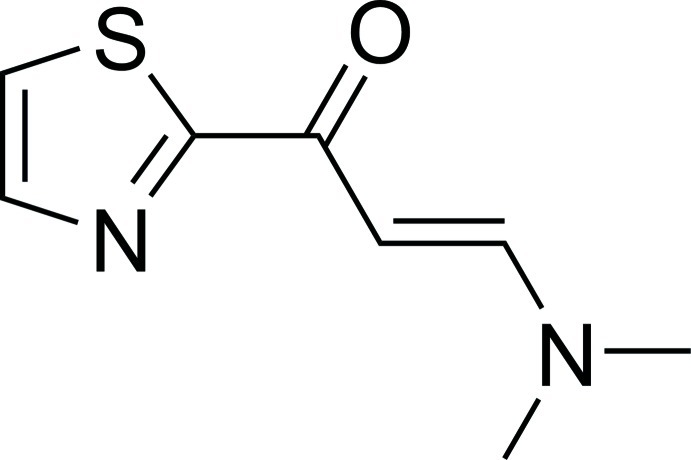


## Experimental

### 

#### Crystal data

C_8_H_10_N_2_OS
*M* *_r_* = 182.24
Monoclinic, 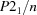

*a* = 5.6252 (2) Å
*b* = 22.5957 (8) Å
*c* = 7.5777 (3) Å
β = 109.498 (4)°
*V* = 907.93 (6) Å^3^
*Z* = 4
Mo *K*α radiation
μ = 0.31 mm^−1^
*T* = 135 K
0.35 × 0.30 × 0.30 mm

#### Data collection

Oxford Diffraction Xcalibur Eos diffractometer
Absorption correction: multi-scan (*CrysAlis PRO*; Oxford Diffraction, 2006 ▶) *T*_min_ = 0.974, *T*_max_ = 1.000
3683 measured reflections
1846 independent reflections
1565 reflections with *I* > 2σ(*I*)
*R*_int_ = 0.018

#### Refinement

*R*[*F* ^2^ > 2σ(*F* ^2^)] = 0.035
*wR*(*F* ^2^) = 0.090
*S* = 1.06
1846 reflections
111 parameters
H-atom parameters constrained
Δρ_max_ = 0.28 e Å^−3^
Δρ_min_ = −0.26 e Å^−3^

### 

Data collection: *CrysAlis PRO* (Oxford Diffraction, 2006 ▶); cell refinement: *CrysAlis PRO*; data reduction: *CrysAlis PRO*; program(s) used to solve structure: *SHELXS97* (Sheldrick, 2008 ▶); program(s) used to refine structure: *SHELXL97* (Sheldrick, 2008 ▶); molecular graphics: *OLEX2* (Dolomanov *et al.*, 2009 ▶) and *Mercury* (Macrae *et al.*, 2006 ▶); software used to prepare material for publication: *OLEX2*.

## Supplementary Material

Crystal structure: contains datablock(s) I, global. DOI: 10.1107/S1600536812048817/vm2183sup1.cif

Structure factors: contains datablock(s) I. DOI: 10.1107/S1600536812048817/vm2183Isup2.hkl

Supplementary material file. DOI: 10.1107/S1600536812048817/vm2183Isup3.cml

Additional supplementary materials:  crystallographic information; 3D view; checkCIF report

## Figures and Tables

**Table 1:** Hydrogen-bond geometry (Å, °) *Cg*1 is the centroid of the S1/N1/C1–C3 ring.

*D*—H⋯*A*	*D*—H	H⋯*A*	*D*⋯*A*	*D*—H⋯*A*
C2—H2⋯N1^i^	0.95	2.62	3.560 (2)	169
C6—H6⋯O1^ii^	0.95	2.60	3.462 (2)	151
C8—H8*A*⋯O1^ii^	0.98	2.31	3.269 (2)	167
C9—H9*C*⋯O1^iii^	0.98	2.51	3.433 (2)	157
C9—H9*A*⋯*Cg*1^iv^	0.98	2.93	3.549 (2)	122

Symmetry codes: (i) 
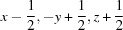
; (ii) 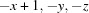; (iii) 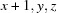; (iv) 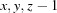.

**Fig. 1.**
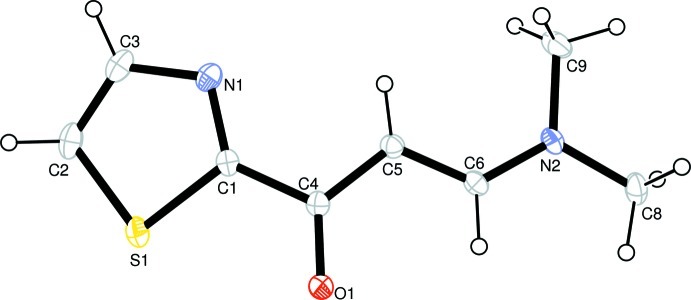
The molecular structure of the title compound, with displacement ellipsoids drawn at the 30% probability level.

**Fig. 2.**
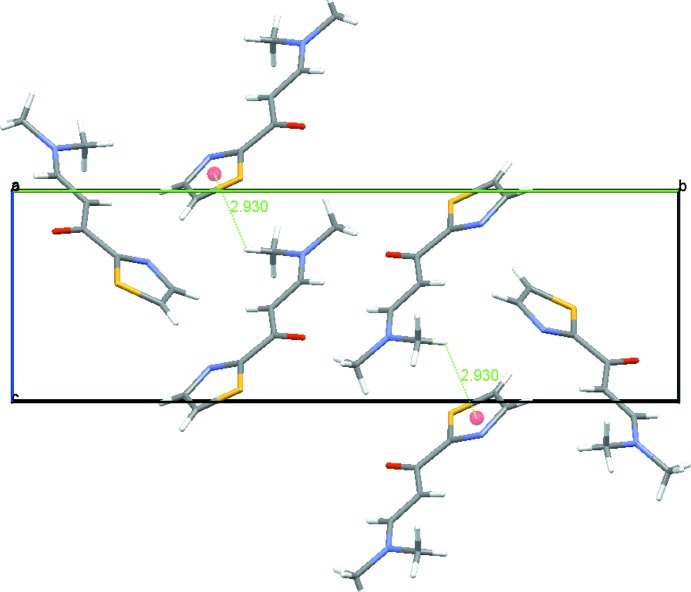
A crystal packing diagram of the title compound. The dashed lines represent C—H···π interactions.

## supplementary crystallographic information

### Comment

Enaminone derivatives are of great importance in organic synthesis (Zeng *et al.*, 2010). The title compound is one of the key intermediates in our synthetic investigations on compounds active against of anti-hepatocellular carcinoma and cancers. We report here its crystal structure.

In the title compound, C_8_H_10_N_2_OS, (Fig.1), the dihedral angle between the thiazole ring and the mean plane through the remaining non-hydrogen atoms [O1/C4 - C9] is 8.88 (3) °. The two double bonds C1═N1 and C4═O1 are in *trans* (N1—C1—C4—O1: 172.31 (15) °), and the C4═O1 and C5═C6 double bonds are in *cis* (O1—C4—C5—C6: 2.2 (2) °).

In the crystal packing (Fig.2), the molecules are stabilized by short intermolecular C—H···N/O interactions and a C9—H9···*Cg*1 interaction (*Cg*1 is the centroid of the S1/N1/C1-C3 ring, Table 1).

### Experimental

A solution of 6.36 g (50.0 mmol) of 1-thiazol-2-yl-ethanone in 16.03 ml (17.87 g,150.0 mmol) of DMF-DMA (dimethoxy-*N*,*N*-dimethylmethanamine) was stirred for 24 h at 114°C. The solvent was evaporated and the title compound was recrystallized from ethanol. Yield: 6.83 g (75%). Crystals suitable for X-ray analysis were obtained by slow evaporation from a solution of ethyl acetate.

### Refinement

All H atoms were positioned geometrically (for methyl, C—H = 0.98 Å; for the other H atoms, C—H = 0.95 Å) and refined using a riding model, with*U*_iso_(H) = k*U*_eq_(C), where k = 1.5 for methyl and 1.2 for all other H atoms.

### Crystal data

**Table:**

C_8_H_10_N_2_OS	*F*(000) = 384
*M**_r_* = 182.24	*D*_x_ = 1.333 Mg m^−^^3^
Monoclinic, *P*2_1_/*n*	Mo *K*α radiation, λ = 0.7107 Å
*a* = 5.6252 (2) Å	Cell parameters from 1788 reflections
*b* = 22.5957 (8) Å	θ = 3.0–28.8°
*c* = 7.5777 (3) Å	µ = 0.31 mm^−^^1^
β = 109.498 (4)°	*T* = 135 K
*V* = 907.93 (6) Å^3^	Block, yellow
*Z* = 4	0.35 × 0.30 × 0.30 mm

### Data collection

**Table:**

Oxford Diffraction Xcalibur Eos diffractometer	1846 independent reflections
Radiation source: Enhanced (Mo) X-ray Source	1565 reflections with *I* > 2σ(*I*)
Graphite monochromator	*R*_int_ = 0.018
Detector resolution: 16.0874 pixels mm^-1^	θ_max_ = 26.4°, θ_min_ = 3.0°
ω scans	*h* = −4→7
Absorption correction: multi-scan (*CrysAlis PRO*; Oxford Diffraction, 2006)	*k* = −13→28
*T*_min_ = 0.974, *T*_max_ = 1.000	*l* = −9→8
3683 measured reflections	

### Refinement

**Table:**

Refinement on *F*^2^	Primary atom site location: structure-invariant direct methods
Least-squares matrix: full	Secondary atom site location: difference Fourier map
*R*[*F*^2^ > 2σ(*F*^2^)] = 0.035	Hydrogen site location: inferred from neighbouring sites
*wR*(*F*^2^) = 0.090	H-atom parameters constrained
*S* = 1.06	*w* = 1/[σ^2^(*F*_o_^2^) + (0.0388*P*)^2^ + 0.2132*P*] where *P* = (*F*_o_^2^ + 2*F*_c_^2^)/3
1846 reflections	(Δ/σ)_max_ < 0.001
111 parameters	Δρ_max_ = 0.28 e Å^−^^3^
0 restraints	Δρ_min_ = −0.26 e Å^−^^3^

### Special details

**Table:**

Experimental. Absorption correction: CrysAlisPro, Agilent Technologies, Version 1.171.35.19, empirical absorption correction using spherical harmonics, implemented in SCALE3 ABSPACK scaling algorithm.
Geometry. All e.s.d.'s (except the e.s.d. in the dihedral angle between two l.s. planes) are estimated using the full covariance matrix. The cell e.s.d.'s are taken into account individually in the estimation of e.s.d.'s in distances, angles and torsion angles; correlations between e.s.d.'s in cell parameters are only used when they are defined by crystal symmetry. An approximate (isotropic) treatment of cell e.s.d.'s is used for estimating e.s.d.'s involving l.s. planes.
Refinement. Refinement of *F*^2^ against ALL reflections. The weighted *R*-factor *wR* and goodness of fit *S* are based on *F*^2^, conventional *R*-factors *R* are based on *F*, with *F* set to zero for negative *F*^2^. The threshold expression of *F*^2^ > σ(*F*^2^) is used only for calculating *R*-factors(gt) *etc*. and is not relevant to the choice of reflections for refinement. *R*-factors based on *F*^2^ are statistically about twice as large as those based on *F*, and *R*- factors based on ALL data will be even larger.

### Fractional atomic coordinates and isotropic or equivalent isotropic displacement parameters (Å^2^)

**Table:**

	*x*	*y*	*z*	*U*_iso_*/*U*_eq_	
S1	0.48299 (8)	0.15794 (2)	0.44117 (6)	0.02965 (16)	
O1	0.5387 (2)	0.06419 (5)	0.19443 (16)	0.0293 (3)	
N1	0.8366 (2)	0.20354 (6)	0.34119 (19)	0.0269 (3)	
N2	0.9036 (2)	0.06400 (6)	−0.19641 (18)	0.0243 (3)	
C1	0.6877 (3)	0.15752 (7)	0.3145 (2)	0.0214 (4)	
C2	0.6091 (3)	0.22388 (9)	0.5379 (2)	0.0347 (4)	
H2	0.5586	0.2455	0.6269	0.042*	
C3	0.7920 (3)	0.24077 (8)	0.4690 (2)	0.0336 (4)	
H3	0.8841	0.2765	0.5073	0.040*	
C4	0.6732 (3)	0.10684 (7)	0.1844 (2)	0.0215 (4)	
C5	0.8103 (3)	0.11210 (7)	0.0581 (2)	0.0226 (4)	
H5	0.9141	0.1456	0.0619	0.027*	
C6	0.7890 (3)	0.06752 (7)	−0.0696 (2)	0.0221 (4)	
H6	0.6800	0.0357	−0.0667	0.026*	
C8	0.8565 (3)	0.01509 (8)	−0.3283 (2)	0.0308 (4)	
H8A	0.7469	−0.0140	−0.2980	0.046*	
H8B	1.0169	−0.0037	−0.3203	0.046*	
H8C	0.7740	0.0300	−0.4556	0.046*	
C9	1.0815 (3)	0.10914 (9)	−0.2102 (2)	0.0342 (4)	
H9A	1.0028	0.1482	−0.2203	0.051*	
H9B	1.1291	0.1017	−0.3214	0.051*	
H9C	1.2324	0.1078	−0.0982	0.051*	

### Atomic displacement parameters (Å^2^)

**Table:**

	*U*^11^	*U*^22^	*U*^33^	*U*^12^	*U*^13^	*U*^23^
S1	0.0328 (3)	0.0320 (3)	0.0283 (3)	0.00526 (19)	0.0159 (2)	−0.00177 (19)
O1	0.0389 (7)	0.0259 (7)	0.0287 (6)	−0.0064 (6)	0.0186 (5)	−0.0032 (5)
N1	0.0274 (7)	0.0248 (8)	0.0253 (7)	0.0021 (6)	0.0046 (6)	−0.0015 (6)
N2	0.0267 (7)	0.0290 (8)	0.0204 (7)	0.0021 (6)	0.0121 (6)	0.0029 (6)
C1	0.0214 (8)	0.0238 (9)	0.0178 (8)	0.0062 (7)	0.0050 (6)	0.0029 (7)
C2	0.0428 (10)	0.0309 (10)	0.0292 (9)	0.0115 (9)	0.0102 (8)	−0.0059 (8)
C3	0.0378 (10)	0.0255 (10)	0.0318 (9)	0.0040 (8)	0.0040 (8)	−0.0064 (8)
C4	0.0232 (8)	0.0223 (8)	0.0179 (8)	0.0028 (7)	0.0052 (6)	0.0026 (7)
C5	0.0238 (8)	0.0235 (9)	0.0209 (8)	−0.0005 (7)	0.0079 (7)	0.0018 (7)
C6	0.0225 (8)	0.0253 (9)	0.0190 (8)	0.0021 (7)	0.0077 (6)	0.0055 (7)
C8	0.0439 (10)	0.0298 (10)	0.0247 (9)	0.0073 (8)	0.0193 (8)	0.0019 (8)
C9	0.0297 (9)	0.0466 (12)	0.0299 (9)	−0.0064 (9)	0.0148 (8)	0.0032 (9)

### Geometric parameters (Å, º)

**Table:**

S1—C1	1.7282 (16)	C3—H3	0.9500
S1—C2	1.707 (2)	C4—C5	1.420 (2)
O1—C4	1.2432 (19)	C5—H5	0.9500
N1—C1	1.308 (2)	C5—C6	1.374 (2)
N1—C3	1.368 (2)	C6—H6	0.9500
N2—C6	1.3264 (19)	C8—H8A	0.9800
N2—C8	1.454 (2)	C8—H8B	0.9800
N2—C9	1.457 (2)	C8—H8C	0.9800
C1—C4	1.495 (2)	C9—H9A	0.9800
C2—H2	0.9500	C9—H9B	0.9800
C2—C3	1.355 (3)	C9—H9C	0.9800
			
C2—S1—C1	89.13 (9)	C6—C5—C4	118.28 (15)
C1—N1—C3	109.85 (14)	C6—C5—H5	120.9
C6—N2—C8	121.51 (14)	N2—C6—C5	127.23 (16)
C6—N2—C9	121.45 (15)	N2—C6—H6	116.4
C8—N2—C9	117.04 (13)	C5—C6—H6	116.4
N1—C1—S1	114.87 (12)	N2—C8—H8A	109.5
N1—C1—C4	127.05 (14)	N2—C8—H8B	109.5
C4—C1—S1	118.07 (12)	N2—C8—H8C	109.5
S1—C2—H2	125.0	H8A—C8—H8B	109.5
C3—C2—S1	109.92 (14)	H8A—C8—H8C	109.5
C3—C2—H2	125.0	H8B—C8—H8C	109.5
N1—C3—H3	121.9	N2—C9—H9A	109.5
C2—C3—N1	116.23 (17)	N2—C9—H9B	109.5
C2—C3—H3	121.9	N2—C9—H9C	109.5
O1—C4—C1	116.99 (14)	H9A—C9—H9B	109.5
O1—C4—C5	125.77 (15)	H9A—C9—H9C	109.5
C5—C4—C1	117.22 (14)	H9B—C9—H9C	109.5
C4—C5—H5	120.9		
			
S1—C1—C4—O1	−9.54 (19)	C1—C4—C5—C6	−176.53 (13)
S1—C1—C4—C5	169.33 (11)	C2—S1—C1—N1	−0.70 (13)
S1—C2—C3—N1	0.2 (2)	C2—S1—C1—C4	−179.08 (13)
O1—C4—C5—C6	2.2 (2)	C3—N1—C1—S1	0.92 (17)
N1—C1—C4—O1	172.31 (15)	C3—N1—C1—C4	179.13 (15)
N1—C1—C4—C5	−8.8 (2)	C4—C5—C6—N2	−179.08 (15)
C1—S1—C2—C3	0.25 (14)	C8—N2—C6—C5	−177.66 (15)
C1—N1—C3—C2	−0.7 (2)	C9—N2—C6—C5	2.1 (3)

### Hydrogen-bond geometry (Å, º)

Cg1 is the centroid of the S1/N1/C1–C3 ring.

**Table:**

*D*—H···*A*	*D*—H	H···*A*	*D*···*A*	*D*—H···*A*
C2—H2···N1^i^	0.95	2.62	3.560 (2)	169
C6—H6···O1^ii^	0.95	2.60	3.462 (2)	151
C8—H8*A*···O1^ii^	0.98	2.31	3.269 (2)	167
C9—H9*C*···O1^iii^	0.98	2.51	3.433 (2)	157
C9—H9*A*···*Cg*1^iv^	0.98	2.93	3.549 (2)	122

Symmetry codes: (i) *x*−1/2, −*y*+1/2, *z*+1/2; (ii) −*x*+1, −*y*, −*z*; (iii) *x*+1, *y*, *z*; (iv) *x*, *y*, *z*−1.
